# Occupational hazard exposures among archivists

**DOI:** 10.3389/fpubh.2025.1631626

**Published:** 2025-07-24

**Authors:** Mei Dou, Xiaomin Wang, Yan Li, Jiaxu Song, Anjing Gong

**Affiliations:** ^1^Department of Neurosurgery, Affiliated Hospital of Qingdao University, Qingdao, China; ^2^Archives, Qingdao University, Qingdao, China; ^3^Institute of Regenerative Medicine and Laboratory Technology Innovation, Qingdao University, Qingdao, China; ^4^Fourth Middle School, Zhangqiu District, Jinan, China; ^5^Qingdao Medical College, Qingdao University, Qingdao, China

**Keywords:** archivists, library, occupational disease, occupational hazards, public health

## Abstract

**Background:**

Archival work environments, often characterized by inadequate ventilation and a high concentration of materials, are prone to the accumulation of diverse harmful substances. Continuous exposure to such an environment may result in an array of health problems.

**Objective:**

This study strives to investigate and synthesize existing research on the occupational hazards encountered by archivists, classify these hazards, detail their associated health impacts, and proffer strategies to preserve and enhance the health of archivists.

**Methods:**

This study followed the PRISMA guidelines to conduct a systematic search of multiple electronic databases, including Web of Science and PubMed, for articles on occupational hazards among archivists. Specific inclusion and exclusion criteria were applied to select relevant studies published between 2000 and 2025. The information organization followed a systematic approach conducted in four stages: question-posing, literature search, literature selection, data extraction and synthesis.

**Result:**

Our review reveals that archivists face multiple occupational hazards. Chemical hazards, including formaldehyde and toluene volatile organic compounds (VOCs), can cause respiratory problems, neurological damage. Biological hazards, such as mold and dust mites, can lead to allergic reactions and respiratory diseases. Physical hazards encompass inadequate temperature and humidity control, insufficient lighting, and ergonomic stress, resulting in eye strain and musculoskeletal injuries. Moreover, overwork and improper postures can cause chronic physical ailments.

**Conclusion:**

This review identifies that archivists are exposed to significant occupational hazards spanning chemical, biological, physical, and ergonomic dimensions, which contribute to a range of health issues. The findings underscore the necessity for in—depth research into archivists’ occupational health and the urgent development of targeted protective strategies to address these hazards.

**Systematic review registration:**

Identifier, CRD420251050852.

## 1 Introduction

Occupational Hygiene is a scientific field focused on predicting, identifying, assessing and controlling health hazards in the workplace, as defined by the International Occupational Health Association (1). While new processes and materials has reduced the presence of many hazardous substances, occupational environments hazards persist and have become more concealed and diverse, leading to chronic health problems and incidenced of disease incidence.

As of the end of 2022, China housed 4,154 archives across various levels, employing a substantial workforce, with 36,582 full-time staff in comprehensive archives alone (Data form National Archives Administration of China). While the precise global number of archivists is challenging to ascertain, it is undoubtedly considerable. In recent years, international awareness of archivists’ occupational health has risen, yet their health risks remain underestimated due to inadequate occupational protection and intricate hazard profiles.

The harmful factors in the archives working environment are complex and diverse, including chemical substances, biological pollutants and physical factors. Long term exposure may lead to a variety of health problems. For example, harmful gases such as formaldehyde and benzene in archives, as well as biological pollutants such as mold and dust mite, may cause allergenic reactions, respiratory diseases, and even carcinogenesis and teratogenesis (2, 3). In addition, archivists also need to face heavy workload and long-term fixed posture in their work, which can easily lead to cervical spondylosis, lumbar spondylosis and other occupational diseases (4).

Therefore, it is crucial to focus on the occupational health of archivists. This review outlines the major health risks for archivists, examines recent studies on chemical, biological, physical hazards and workload, notes gaps in understanding these risks, and stresses the urgent need for effective preventive and control measures to protect archivists’ the health and safety.

## 2 Methods

The review was conducted in four stages: question-posing, literature search, literature selection, data extraction and synthesis. Due to significant heterogeneity in the included studies’ research designs, outcome measurements, and variable definitions, as well as differences in risk assessment methods and sample geographical backgrounds, meta—analysis may not yield accurate and representative results. Therefore, we used a narrative synthesis approach to summarize and analyze the study results.

### 2.1 Question-posing

Archivists face diverse occupational hazards in closed work settings. Their health has received little attention in occupational health research. Existing studies usually focus on single hazard factors, lacking a comprehensive analysis of all related occupational health hazards. Moreover, methodological differences among studies have impaired result comparability and consistency. Thus, a more comprehensive review of archivists’ occupational health hazards is urgently needed.

### 2.2 Literature search

#### 2.2.1 Protocols

Participants, interventions, comparisons, outcomes and study design (PICOS) and Preferred Reporting Items for Systematic Reviews and Meta-Analyses (PRISMA) protocol was used.

In our review, we applied the PICOS framework as follows:

Population: Archivists.

Intervention: Occupational exposure to chemical, biological, physical factors and workload, etc.

Comparison: No specific control group, mainly comparing exposed and non-exposed situations.

Outcome: Occupational health status of archival staff, including biological, chemical, physical, and overwork factors.

Study design: Including clinical trials, original research and cohort study etc., excluding meta-analyses, reviews, case reports, etc.

#### 2.2.2 Databases and searched strategies

PubMed and Web of Science were employed with Boolean Logic (Tables 1, 2) for the literature search from January 1, 2000, to January 1, 2025.

**Table 1:** PubMed search strategy.

Group	Search strategy
#1	Library[MeSH] OR Librarian[All Fields] OR Archivist[All Fields]
#2	Organic Chemicals[MeSH] OR Benzene[MeSH] OR Formaldehyde[MeSH] OR Toluene[MeSH] OR Xylenes[MeSH] OR Nitrogen Oxides[MeSH] OR Sulfur Dioxide[MeSH] OR Fluorides[MeSH] OR Hydrogen Sulfide[MeSH] OR Ammonia[MeSH] OR Carbon Monoxide[MeSH] OR Asbestos[MeSH] OR Styrene[MeSH]
#3	Microbiology[MeSH] OR Biohazards[MeSH] OR Allergens[MeSH] OR Fungi[MeSH] OR Bacteria[MeSH] OR Virus[MeSH]
#4	Dust[MeSH] OR Electromagnetic Radiation[MeSH] OR Ultraviolet Rays[MeSH] OR Temperature[MeSH] OR Humidity[MeSH] OR Air[MeSH] OR Light[MeSH]
#5	Burnout, Professional[MeSH] OR Workload[MeSH]
#6	(# 2) OR (# 3) OR (# 4) OR (# 5)
Final search strategy	(# 1) AND (# 6)

**Table 2:** Web of science search strategy.

Group	Search strategy
#1	TS = (Librarian)) OR TS = (Archivist)
#2	((((((((((((TS = (Organic Chemicals)) OR TS = (Benzene)) OR TS = (Formaldehyde)) OR TS = (Toluene)) OR TS = (Xylenes)) OR TS = (Nitrogen Oxides)) OR TS = (Sulfur Dioxide)) OR TS = (Fluorides)) OR TS = (Hydrogen Sulfide)) OR TS = (Ammonia)) OR TS = (Carbon Monoxide)) OR TS = (Asbestos)) OR TS = (Styrene)
#3	(((((TS = (Microbiology)) OR TS = (Biohazards)) OR TS = (Allergens)) OR TS = (Fungi)) OR TS = (Bacteria)) OR TS = (Virus)
#4	((((((TS = (Dust)) OR TS = (Electromagnetic Radiation)) OR TS = (Ultraviolet Rays)) OR TS = (Temperature)) OR TS = (Humidity)) OR TS = (Air)) OR TS = (Light)
#5	(TS = (Burnout)) OR TS = (Workload)
#6	(((#2) OR #3) OR #4) OR #5
Final search strategy	(#1) AND #6

### 2.3 Literature selection

We used the PRISMA protocol and applied inclusion/exclusion criteria for literature selection.

Authors involvement: Mei Dou, Xiaomin Wang, Jiaxu Song, and Anjing Gong.

Inclusion of the review: Studies on archivists’ occupational health, hazards, and exposure involve four areas: biological, chemical, physical, and overwork factors.

Exclusion of the review: Studies unrelated to archivists, lacking primary data, or irrelevant to occupational health. Also excluded were those not covering the four areas of biological, chemical, physical, and overwork factors, but focusing instead on mechanical, psychological, electrical, and ergonomic hazards. This is due to the insufficient number of such studies for a comprehensive review.

### 2.4 Data extraction and synthesis

Authors involvement: Mei Dou, Xiaomin Wang, Yan Li, and Anjing Gong.

Data extraction was performed independently by two authors (Mei Dou and Xiaomin Wang) using a standardized data extraction form. The following information was extracted from each included study: consisted authors, design used, number of population (archivists), main outcome, suggested mitigation and types of occupational hazards assessed (including biological, chemical, physical, and overwork factors). Any discrepancies were resolved by discussion with the other authors (Yan Li and Anjing Gong). The extracted data were then synthesized and analyzed to identify common themes, patterns, and gaps in the existing literature.

## 3 Results

### 3.1 Study selection

**Figure 1:**
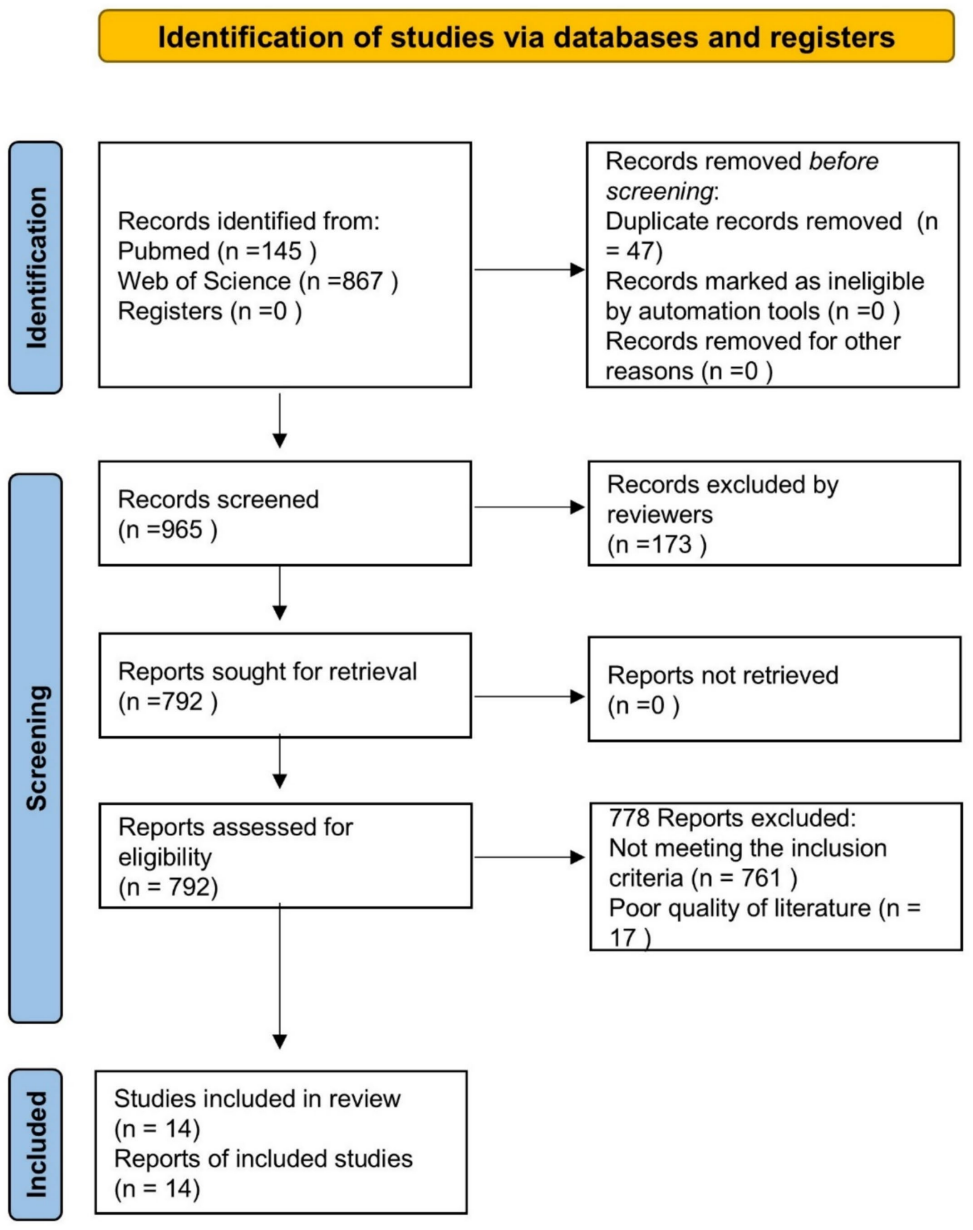
PRISMA flow diagram.

We identified 1,012 records through the search process based on the PRISMA flowchart. After removing 47 records duplicates and 173 records that not meet the type of study, we screened 792 records based on titles and abstracts. Subsequently, we assessed 14 studies in full text and included 778 in our review (Figure 1).

### 3.2 Study characteristics

Table 3 summarises the included studies’ characteristics. These studies cover 14 countries: China, Poland, Thailand, USA, Pakistan, U.K., Ireland, South Korea, Cyprus, Italy, Cuba, Colombia, France, and Iran. They use diverse designs: cross-sectional, preliminary investigations, original research, questionnaires, perspectives and initiatives, and comparative studies. Most participants are archivists, though some studies do not specify the number. The focus is on exposure to pollutants in these settings, such as inhalable particles (PM2.5, PM4, PM10), CO₂, bacteria, fungi, and VOCs. Health impacts on staff, including asthma, COVID-19 exposure, and decision fatigue from overwork, are also studied. To address these issues, the studies recommend various measures: environmental condition monitoring and control, and enhanced protective measures.

**Table 3:** Study characteristics.

Hazards type	Specific Hazards	Country	Design	Suggested mitigation	Authors	Ref.
Chemical hazards	Content of VOCs	U. K. and Ireland	Original Research	N/A	Gibson et al.	(7)
Chemical hazards	Content of indoor Air Pollutants	Korea	Original Research	High concentrations of individual VOCs, aldehydes, and organic acids were detected, and additional measures are required to manage these substances.	Lee et al.	(9)
Chemical hazards	Content of VOCs	Cyprus	Original Research	Control storage conditions to ensure a safe, contamination—free environment for books and readers.	Stylianou et al.	(20)
Chemical hazards	Content of PM2.5, NO2, SO2, O3	Italy	Original Research	The situation can be greatly improved by monitoring and controlling the ambient conditions.	Cappitelli et al.	(24)
Chemical hazards, biological hazards	Content of air-and dustborne fungi	Cuba	Original Research	N/A	Borrego et al.	(27)
Biological hazards	Content of Microbiological contamination with fungi, including moulds	Poland	Original Research	N/A	Zielinska-Jankiewicz et al.	(28)
Biological hazards	Content of fungi	Colombia	Original Research	Caution measurements are suggested to be reinforced in archives for appropriate workers protection	Castillo et al.	(29)
Biological hazards	Content of fungi	France	Original Research	N/A	Roussel et al.	(32)
Biological Hazards	Content of fungi	Iran	Comparative Study	N/A	Foladi et al.	(34)
Biological hazards	Exposure to fungi	Poland	Preliminary investigation	N/A	Cyprowski et al.	(52)
Biological hazards, physical hazard, chemical hazards	Exposure to respirable dust, PM2.5, PM4, PM10, CO2, bacteria, fungi, air temperature, %RH, and air movement	Thailand	Original Research	Photocopy shop in Library should undergo regular cleaning procedures and maintenance of air conditioning systems to achieve goodindoor air quality	Limmongkon et al.	(49)
Overwork	Decision fatigue	USA	Questionnaire	When the decisions are complex or involve a large volume of information, then employing a flowchart, framework, or some other means may simplify processes	Natal and Saltzman	(50)
Others	Asthma attacks	China	Cross-sectional study	N/A	Yang et al.	(47)
Others	Exposure to COVID-19	Pakistan	Perspectives and Initiatives	Support public health awareness; support research teams, researchers and faculty; and provide routine core services for regular library users	Ali and Gatiti	(48)

### 3.3 Study outcomes

#### 3.3.1 Chemical hazards

##### 3.3.1.1 VOC

Archivists are primarily at risk from volatile organic compounds (VOCs) released by building and archival materials (5). Archives are typically kept in enclosed environments to protect their holdings. However, a poorly designed or maintained ventilation system can limit air exchange with the outside, allowing VOCs and other pollutants to accumulate and exacerbate indoor air pollution (6).

Cases from Portugal and the United Kingdom have shown that concentrations of VOCs, such as toluene and acetaldehyde, in archives often exceed legal limits. This may result from the degradation of paper materials, the release of chemicals from building renovation materials, and inadequate ventilation (7, 8). While the total VOCs (TVOCs) in the National Library of Korea meet the required standard, the high percentage of toluene and acetaldehyde indicates that the toxic effects of specific compounds warrant attention (9). Benzene-related compounds (benzene, toluene, and xylene) are particularly prevalent in Chinese homes, offices, and schools, often linked to poor-quality building materials and intensive renovations (10). Although routine indoor environments typically have low VOC concentrations, making it unlikely for these to reach carcinogenic thresholds, sites like archives can experience long-lasting, abnormal peaks in VOC levels due to airtightness, material aging, or external pollutant inputs. This can lead to significant cumulative exposure for staff members (11).

During renovation, building materials, decorations, and furniture release VOCs like formaldehyde, benzene, toluene, and xylene (12). Similarly, archival materials emit various VOCs, including the same compounds, during long—term storage. These substances are volatile and pose potential health risks. Among these, toluene and formaldehyde are the most concentrated VOCs, released from paper, ink, adhesives, etc., under certain conditions.

Toluene, a colorless, volatile liquid with a distinct aromatic odor, is insoluble in water but miscible with ethanol, ether, and other organic solvents. Long—term exposure can damage the nervous system, causing headaches, dizziness, and lack of concentration, as well as respiratory issues and potentially increasing cancer risk (13, 14). Formaldehyde, a colorless gas with a pungent odor, is highly soluble in water, alcohol, and ether. It is a proven carcinogen with a strong irritant effect on the eyes, nose, throat, and other mucous membranes (15). Prolonged exposure to low—dose formaldehyde can harm the central nervous system and liver function, potentially causing cancer, cardiovascular disease, allergic rhinitis, etc. (16, 17). Concentrations exceeding 0.5 mg/m^3^ may lead to miscarriages in pregnant women and leukemia in infants (18). Besides, long-term exposure to low-dose organic solvents can harm the central nervous system and liver function, causing cancer cardiovascular disease, allergenic rhinitis, etc. (19).

These chemical contaminants pose dual threat: to the health of archives staff and to the integrity of paper materials, accelerating their aging and damage, which manifests as handwriting fading and paper brittleness (20). To mitigate these risks, archives should implement adopt effective ventilation measures, conduct regular air quality monitoring and control within the facilities, air quality and utilize environmentally friendly materials and protection technologies. These measures can reduce the generation and impact of chemical pollutants, ensuring both the preservation of archival materials and the health of staff.

##### 3.3.1.2 Particulate matter 2.5

Particulate matter 2.5 (PM2.5) refers to particulate matter in the air that is less than or equal to 2.5 microns in diameter. The World Health Organzation listed polluted air containing PM2.5 as a class I carcinogen (21). There is a large amount of PM2.5 in the archives, because these particles are very small, they can be suspended in the air for a long time and can be inhaled by the human body, causing pulmonary and heart diseases (22). Long-term exposure enhances allergenic sensitization, exacerbates asthma symptoms, and induces various cancers (23).

In an assessment of indoor air quality in the Ca’ Granda Historical Archive, the composition of PM2.5 was found to be dominated by organic carbon, likely due to the presence of archival materials made of paper (24). Furthermore, SO4^2−^ and NH4^+^ are also important contributors to indoor PM2.5, occupying significant proportions in the main hall and basement, respectively (25). OC has been found to contain many toxic substances, including polycyclic aromatic hydrocarbons, organic acids, n-alkanes, and persistent organic pollutants. These components are potentially teratogenic, mutagenic, and carcinogenic, which can easily increase the risk for human diseases (26). Therefore, archivists are exposed to PM for a long time and need to take effective protective measures to reduce PM exposure and health effects.

#### 3.3.2 Biological hazards

The health of archivists is also affected by a number of biological factors, particularly the overabundance of microbes and dust mites in archives.

##### 3.3.2.1 Microbes

The presence of microorganisms is a common phenomenon in the archive environment, especially molds among fungi (27). These microorganisms are not only widely distributed inside archives, but are also common in outdoor environments. They can be accessed in a variety of ways, including doorways, windows, ventilation, air-conditioning, heating systems, and the movement of workers and archive entities. Zielinska-Jankiewicz et al. (28) assessed the health hazards of archivists exposed to mold pollution and found that12 species considered potentially pathogenic to humans. Samples of broths of fungal species isolated from documentary material, and indoor environmental samples from the Archive of Bogotá, identified up to 44 mycotoxins, 34 of which were confirmed by subsequent analysis (29). The presence of mold in the archives environment and the toxins it produces pose serious challenges to archives protection and personnel health.

Most molds are allergenic, toxicogenic, or infectious to humans and could cause discomforting symptoms (30). A sufficiently high mold concentration in the air can lead to mold inhalation (31). This could cause fungal infection in the respiratory tract especially in cases of low immunity. Contact with moldy papers was significantly associated with high incidences of headache, fatigue, eye irritation, throat irritation, coughing, and nasal bleeding (32). Some data suggest that airborne fungal concentrations in certain repositories (e.g., the National Archives of the Republic of Cuba) indicate an uncontaminated environment (33). However, other data show the opposite result, suggesting that high airborne fungal concentrations may increase the risk of respiratory diseases among staff (34). Therefore, effective preventive and control measures to reduce mold contamination and toxin production are essential to protect archival materials and ensure staff health.

##### 3.3.2.2 Dust mites

Dust mites are a class of tiny arthropods that are particularly common in indoor environments, especially in enclosed and dusty spaces such as archives. Allergens in dust mites and their excretions are the main causes of allergenic diseases, including asthma, allergenic rhinitis and atopic dermatitis (35). Among archivists, dust mite allergen levels did not always show a direct correlation with the number of live mites in indoor dust. This is because even dead or decomposed dust mites are still allergenic. These allergens can be transmitted through the air, and when people perform indoor activities, they may inhale these allergens, which can trigger allergenic reactions (36).

A study of occupational respiratory allergy symptoms conducted by Corrao et al. (37), 7 of 21 file workers surveyed tested positive for dust mites, a positive rate of 17.1%. This percentage is significantly higher than in other occupational groups. This finding suggests that the presence of dust mite allergens in the archive environment poses a potential health threat to archivists. To reduce exposure to dust mite allergens, effective indoor environmental control measures such as regular cleaning, improved ventilation, and the use of dust mite control products are required. In addition, regular health monitoring and allergen testing of archivists is necessary to detect and address allergies in a timely manner.

#### 3.3.3 Physical hazard

Archivists are often exposed to a variety of physical factors in their daily work that may adversely affect their health. Physical hazards include, but are not limited to, solid particles, ventilation, electromagnetic radiation, temperature and humidity, insufficient light, etc. Physical factors and other biochemical factors interact to increase the health risks of archive staff.

##### 3.3.3.1 Temperature and humidity

In the field of archives protection, the control of temperature and humidity is very important for the long-term preservation of material. Archives usually have strict temperature and humidity standards to ensure the stability and integrity of archival materials (38). The Archives Industry Standard of the People’s Republic of China stipulates that the temperature and humidity of the archives warehouse should be maintained relatively stable, with the daily temperature range ≤ ± 2°C and the daily humidity range ≤ ± 5%. Improper temperature and humidity control may lead to accelerated reproduction of microorganisms and mites (39). At certain concentrations, these organisms may not only damage archival materials, but may also pose a health threat to archivists, causing allergenic reactions, respiratory irritation or other health problems. Exposure to low relative humidity (RH) can lead to the deterioration of tear film stability, resulting in intraocular dryness, increased osmolarity, and inflammatory responses. Dry air exposure may also increase peripheral airway resistance, triggering airway constriction and reducing mucosal ciliary clearance time, thereby impairing the body’s defense mechanisms against influenza viruses (40). Therefore, the temperature and humidity control strategy of archives must take into account the inhibition of microbial ecology and the need for health protection of archivists.

##### 3.3.3.2 Light

Light is also an important factor affecting the health of archivists, and the storage and use areas of archives are usually kept in dark conditions to maintain the physical state of the archives. However, long-term work in low-light environments can cause eye fatigue, soreness and blurred vision (41). Pupil dilation in the dark may gradually induce glaucoma risk in people with shallow anterior chambers (42). Therefore, the thoughtful design of the lighting system in the archival work environment, along with the implementation of scheduled eye breaks, has become essential in balancing the preservation of archives with the health and well-being of staff.

##### 3.3.3.3 Security risks

In their daily work, archivists also face obvious security risks in the working environment, especially in the process of file storage, handling and file management. The risk of falling is common in stairs and high storage areas, and staff are prone to slip or fall injured when taking high files or unstable ladders (43). In addition, the handling and storage of files involves frequent lifting movements, which can easily lead to muscle strains or spinal injuries. Ergonomic problems can not be ignored, long standing, bending and repetitive movements may lead to cervical spondylosis, lumbar disease and other occupational diseases (44). Another common hazard is being injured by falling books or files, and heavy paper files can cause serious immediate injury to staff.

In addition to the above factors, there are other physical factors that may affect the health of archivists, although these effects are relatively small, but equally worthy of attention. Archives management agencies should adopt a combination of measures, including improved temperature and humidity control, reduced exposure to electromagnetic radiation, provision of appropriate lighting conditions, and increased awareness of health protection among archivists, to ensure the health and safety of records and staff.

#### 3.3.4 Overwork

Excessive workloads may cause musculoskeletal injuries such as lower back, cervical, knee, and shoulder pains, due to unnatural postures or repetitive and rapid movements (45). When engaged in archival work such as collecting, organizing, or restoring, archivists often have to sit unnaturally for long periods, leading to injuries. Previous studies reported on the physical sub-health states of workers, manifested as chronic fatigue and varying degrees of cervical spine, shoulder circumference, lumbar spine, and wrist pain (46).

#### 3.3.5 Others

Some other factors that can harm the health of archivists should not be ignored, either. Asthma, a chronic airway inflammatory disease caused by multiple factors, is a case in point. It often results from the interplay of various internal and external factors. For archivists, while certain biological and chemical substances in the workplace (e.g., dust, mold spores, cleaner fumes) may trigger asthma, individual health, genetics, lifestyle (smoking, exercise) and stress also play a role. So, it’s not accurate to classify it simply as a biological or chemical risk factor (47). COVID-19, a global public health emergency, is another example. The virus is not a inherent biological risk in the library and archives environment. Instead, it’s a special external risk affecting this particular workplace, making it hard to classify it as a traditional biological risk factor (48).

## 4 Discussion

This review sheds light on the critical yet often overlooked issue of occupational health and safety for archivists, who face a range of hazards from chemical exposures (e.g., formaldehyde, toluene), biological contaminants (e.g., mold, dust mites), and physical risks such as extreme temperatures, humidity fluctuations, poor lighting, and ergonomic strain. These risks are amplified by a complex interplay of environmental factors—poor ventilation increases the concentration of indoor pollutants, while inadequate temperature and humidity control fosters microbial growth, creating a harmful cycle that endangers both archival materials and worker health. Additionally, other factors such as asthma attacks and COVID-19 exposure also pose significant health risks to archivists. These hazards are not inherent biological risks within the library or archival environment itself. Instead, they result from the combined effects of individual genetic susceptibility and external environmental factors. Therefore, they cannot be fully categorized under traditional biological risk classifications.

Due to significant heterogeneity in study designs, outcome measurements, and variable definitions, quantitative synthesis was limited and meta—analysis wasn’t used. However, we still adhered to the rigor of the scientific method in our systematic review.

Although each of these hazards presents a potential threat to the health of archivists, there are significant differences in their associated risk levels. Research indicates that chemical exposures pose the greatest occupational health risks due to their mutagenic, carcinogenic, and chronic toxic effects. This is further supported by numerous studies conducted worldwide on these hazards (7, 9, 20, 24, 49). Biological contamination also presents cumulative risks, but its pathogenic mechanisms are relatively well-understood, and effective prevention and control measures are in place. Relevant research primarily focuses on air microbial monitoring and the enhancement of personal protective equipment (29, 32, 34). Physical factors such as abnormal temperature and humidity, insufficient lighting, etc. are more potential factors of occupational hazards, and the existing cases are mostly synergistic with other chemical/biological factors, which indirectly increase the risk of exposure to other hazards (50).

Based on the above occupational hazard risks for archivists, we present specific, actionable recommendations grounded in methodology. To accurately quantify chemical exposure risks, a prospective cohort study should be conducted in archival repositories, tracking formaldehyde/toluene exposure using individualized VOC monitoring equipment (e.g., passive samplers or real-time PID detectors) to assess risks at the individual level. For biological hazards, the effectiveness of HEPA filtration systems in controlling airborne fungal spore concentrations should be evaluated, and changes in staff allergen-specific IgE levels should be monitored. Physical hazards should be examined using ergonomic assessment tools (e.g., OWAS postural analysis) to develop standardized file-handling procedures and quantify postural loads. Interventions for overwork-related injuries should validate the preventive effect of intermittent micro-break regimes on musculoskeletal disorders.

As a relatively small and specialized group, archivists have not received the attention they deserve in discussions of occupational health and safety, and their specific challenges are often overlooked. This review provides a comprehensive examination of the working environment for archivists and the potential physical harm they may encounter, considering the issue from multiple perspectives. It advocates for increased awareness of the health risks faced by archivists and offers a theoretical foundation for the development of relevant policy standards.

For policymakers, the findings underscore the urgent need for reforms, including stricter enforcement of ventilation standards, mandatory air quality monitoring, and the integration of ergonomic practices into occupational safety frameworks—measures that are largely absent from current regulations. Institutions must evolve beyond a sole focus on preserving materials to also prioritize staff well-being by improving workplace design, using environmentally friendly materials, and implementing routine health screenings. Furthermore, archivists should have access to enhanced protective equipment, proper training, and better systems support to minimize risks and improve their overall quality of work life (51).

## 5 Conclusion

Archivists face a range of occupational hazards, and it is essential to prioritize interventions based on the severity and prevalence of risks identified in existing literature. Chemical hazards, especially carcinogenic volatile organic compounds (VOCs) like formaldehyde and toluene released from aging materials, pose significant threats to both human health and the integrity of archival materials. Biological risks, including mold and dust mites, are equally critical, as their allergens and toxins can lead to respiratory issues, allergies, and more severe systemic health problems. Furthermore, physical hazards, such as inadequate temperature and humidity control, poor lighting, and ergonomic risks, can compound health concerns by causing eyestrain and increasing musculoskeletal injuries. Chronic physical ailments among archivists, resulting from overexertion due to prolonged static postures and repetitive movements, also require attention. To mitigate these risks, we recommend mandating the use of volatile organic compound (VOC) detectors in archive rooms. Additionally, archivists should undergo comprehensive occupational health screenings at least once a year. Regular cleaning and the installation of air filtration systems are essential to reduce exposure to mold and dust mites. These measures are designed to promote a healthier and more sustainable working environment.

## 6 Strengths and limitations

This narrative review offers a comprehensive examination of the occupational hazards faced by archivists, integrating interdisciplinary evidence to address the complex nature of chemical, biological, physical, and ergonomic risks. Its strength lies in the flexibility of the narrative approach, which enables a holistic analysis of interacting hazards, such as the synergistic effects of chemical and biological exposures. The review also systematically incorporates literature from various databases (e.g., Web of Science, PubMed) and secondary sources to compensate for the lack of direct empirical research.

However, it is important to acknowledge the inherent methodological limitations of narrative reviews. These include the potential subjectivity in literature selection and the inclusion of non-primary studies (such as reviews) to ensure a broad coverage of the subject matter, which may compromise the robustness of the evidence. Additionally, the limited number of experimental or cohort studies specifically focused on the occupational health of archivists calls for caution when interpreting some of the conclusions derived from theoretical extrapolations.

In addition, several specific limitations of this review merit consideration. First, the potential for publication bias cannot be excluded. Studies reporting significant findings related to occupational hazards or adverse health effects were more likely to be published than those without significant associations, which may have led to an overestimation of risk in the reviewed literature. Second, our search strategy was confined to articles published in English. Although this is a standard practice in systematic reviews, it inevitably excludes relevant studies published in other languages, which may introduce language bias and result in the omission of valuable data and perspectives. Third, although the included studies span 14 countries, geographic representation remains limited. The majority of detailed studies on chemical and biological hazards originated from Europe, East Asia, and North America. This geographic limitation reduces the generalizability of our findings to all archival settings worldwide, as hazard profiles, exposure levels, occupational safety regulations, and available resources for hazard mitigation can vary considerably across different socioeconomic and climatic regions.

## Data Availability

The original contributions presented in the study are included in the article/supplementary material, further inquiries can be directed to the corresponding author.
