# Correction: Occupational hazard exposures among archivists

**DOI:** 10.3389/fpubh.2025.1673563

**Published:** 2025-08-29

**Authors:** Mei Dou, Xiaomin Wang, Yan Li, Jiaxu Song, Anjing Gong

**Affiliations:** ^1^Department of Neurosurgery, Affiliated Hospital of Qingdao University, Qingdao, China; ^2^Archives, Qingdao University, Qingdao, China; ^3^Institute of Regenerative Medicine and Laboratory Technology Innovation, Qingdao University, Qingdao, China; ^4^Fourth Middle School, Zhangqiu District, Jinan, China; ^5^Qingdao Medical College, Qingdao University, Qingdao, China

**Keywords:** archivists, library, occupational disease, occupational hazards, public health

In the published article, there was an error in affiliation [3]. Instead of “[Fourth Middle School, Jinan, China]”, it should be “[Fourth middle school, Zhangqiu District, Jinan, China]”.

Affiliation “[Department of Neurosurgery, Affiliated Hospital of Qingdao University, Qingdao, China]” of the corresponding author “[Anjing Gong]” should be listed as affiliation [1], with all other affiliations following in sequence.

Such as:

Mei Dou^2†^, Xiaomin Wang^3†^, Yan Li^4^, Jiaxu Song^5^ and Anjing Gong^1*^

^1^Department of Neurosurgery, Affiliated Hospital of Qingdao University, Qingdao, China

^2^Archives, Qingdao University, Qingdao, China

^3^Institute of Regenerative Medicine and Laboratory Technology Innovation, Qingdao University, Qingdao, China

^4^Fourth Middle School, Zhangqiu District, Jinan, China

^5^Qingdao Medical College, Qingdao University, Qingdao, China

The original article has been updated.

